# Xylose fermentation efficiency of industrial *Saccharomyces cerevisiae* yeast with separate or combined xylose reductase/xylitol dehydrogenase and xylose isomerase pathways

**DOI:** 10.1186/s13068-019-1360-8

**Published:** 2019-01-28

**Authors:** Joana T. Cunha, Pedro O. Soares, Aloia Romaní, Johan M. Thevelein, Lucília Domingues

**Affiliations:** 1 CEB-Centre of Biological Engineering, University of Minho, 4710-057 Braga, Portugal; 2 Laboratory of Molecular Cell Biology, Institute of Botany and Microbiology, Department of Biology, KU Leuven, Kasteelpark Arenberg 31, 3001 Leuven-Heverlee, Flanders Belgium; 3 Center for Microbiology, VIB, Kasteelpark Arenberg 31, 3001 Leuven-Heverlee, Flanders Belgium

**Keywords:** Hemicellulosic ethanol, Xylose consumption, Industrial yeast, Xylose isomerase, Xylose reductase/xylitol dehydrogenase, Lignocellulosic hydrolysates

## Abstract

**Background:**

Xylose isomerase (XI) and xylose reductase/xylitol dehydrogenase (XR/XDH) pathways have been extensively used to confer xylose assimilation capacity to *Saccharomyces cerevisiae* and tackle one of the major bottlenecks in the attainment of economically viable lignocellulosic ethanol production. Nevertheless, there is a lack of studies comparing the efficiency of those pathways both separately and combined. In this work, the XI and/or XR/XDH pathways were introduced into two robust industrial *S. cerevisiae* strains, evaluated in synthetic media and corn cob hemicellulosic hydrolysate and the results were correlated with the differential enzyme activities found in the xylose-pathway engineered strains.

**Results:**

The sole expression of XI was found to increase the fermentative capacity of both strains in synthetic media at 30 °C and 40 °C: decreasing xylitol accumulation and improving xylose consumption and ethanol production. Similar results were observed in fermentations of detoxified hydrolysate. However, in the presence of lignocellulosic-derived inhibitors, a positive synergistic effect resulted from the expression of both XI and XR/XDH, possibly caused by a cofactor equilibrium between the XDH and furan detoxifying enzymes, increasing the ethanol yield by more than 38%.

**Conclusions:**

This study clearly shows an advantage of using the XI from *Clostridium phytofermentans* to attain high ethanol productivities and yields from xylose. Furthermore, and for the first time, the simultaneous utilization of XR/XDH and XI pathways was compared to the single expression of XR/XDH or XI and was found to improve ethanol production from non-detoxified hemicellulosic hydrolysates. These results extend the knowledge regarding *S. cerevisiae* xylose assimilation metabolism and pave the way for the construction of more efficient strains for use in lignocellulosic industrial processes.

## Background

The depletion of fossil fuel reserves, the economic problems associated with their use and the growing environmental concerns related with greenhouse gas emissions have led to a search for new renewable energy sources [1]. The use of lignocellulosic biomass for the production of bioethanol and value-added products has emerged as a sustainable alternative to fossil sources, as lignocellulose is one of the most abundantly available renewable biomass sources on earth and its utilization does not compete with the use of land for food production [2–4]. The complex and recalcitrant structure of lignocellulosic biomass comprises cellulose (a crystalline, linear homopolymer of glucose), hemicellulose (a branched, amorphous heteropolymer of hexoses and pentoses) and lignin [1]. The pre-treatment and hydrolysis steps, required to obtain fermentable sugars from lignocellulosic biomass, also release inhibitory compounds, such as 5-hydroxymethylfurfural (HMF), furfural and acetic acid [5]. These compounds strongly affect microbial growth and ethanol fermentation [6, 7]. Glucose and xylose are the most abundant monosaccharides present in lignocellulosic hydrolysates, representing between 60–70% and 30–40% of their sugar composition, respectively [8].

*Saccharomyces cerevisiae*, the preferred microorganism for large-scale ethanol production, does not naturally consume xylose. Thus, to obtain economically viable production of bioethanol, the hemicellulosic fraction composed mainly of xylose should be efficiently converted into ethanol through development of a *S. cerevisiae* strain capable of consuming xylose as well as having strong resistance to inhibitory compounds. Xylose assimilation is achieved through conversion of xylose into xylulose and subsequent phosphorylation to xylulose-5-phosphate, which is further metabolized in the pentose phosphate pathway. Two different pathways have previously been expressed in *S. cerevisiae* to convert xylose into xylulose: the oxidoreductase and the isomerase pathway. The oxidoreductase pathway is used by many xylose-fermenting yeast species and consists of two enzymatic reactions catalyzed by xylose reductase (XR) and xylitol dehydrogenase (XDH) [9]. First, XR reduces xylose to xylitol, preferably using NADPH over NADH as cofactor; xylitol is then oxidized to xylulose by XDH, which uses only NAD^+^ as cofactor. The XR/XDH pathway has a bottleneck caused by a cofactor imbalance between the mainly NADPH-dependent XR and the NAD^+^-dependent XDH, which generally causes xylitol accumulation and thus lowers ethanol production [10]. Additionally, *S. cerevisiae* carries the endogenous gene *GRE3* that encodes an unspecific NADPH-dependent aldose reductase that can convert xylose to xylitol [11]. The expression of this enzyme, which only uses NADPH as cofactor, might aggravate the redox imbalance, therefore leading to even greater xylitol accumulation and to inefficient xylose fermentation [12]. In this sense, the deletion of *GRE3* [13–15] and genetic modifications supporting cofactor regeneration [16] decrease xylitol accumulation and consequently increase ethanol production.

The isomerase pathway is mainly found in bacteria and is a one-step reaction catalyzed by xylose isomerase (XI) that directly converts xylose to xylulose without cofactor requirement [8, 17]. Initial attempts to express bacterial xylose isomerase in yeast were not very successful, except for a xylose isomerase from a thermotolerant bacterium *Thermus thermophilus* [18]. Expression of a xylose isomerase from the fungus *Piromyces* sp. resulted for the first time in efficient xylose fermentation with this pathway [19]. Later, Brat et al. [20] were able to express a highly efficient xylose isomerase from *Clostridium phytofermentans* in a laboratory *S. cerevisiae* strain, also resulting in efficient xylose fermentation. Functional expression of bacterial xylose isomerase in an industrial *S. cerevisiae* strain was more challenging and was accomplished only after extensive mutagenesis and evolutionary engineering [21]. Increasing inhibitor tolerance further improved the performance of this industrial yeast strain [22]. Because of the inhibitory effect of xylitol on XI activity [23], the strategies used to reduce xylitol production in XR/XDH strains can also improve the isomerization activity of XI in vivo and thus also improve xylose fermentation rate in strains with XI [24].

Industrial environments have been a resource of robust *S. cerevisiae* strains, exhibiting higher fermentation performance and resistance to lignocellulosic-derived inhibitors when compared to other laboratory strains [25]. Thermotolerance is another trait presented by some yeast strains isolated from industrial environments that can be desirable for bioethanol fermentation. As previously mentioned, an economically viable production of second-generation ethanol passes by an efficient fermentation of the lignocellulosic-derived whole slurry (cellulosic and hemicellulosic fractions), and in this sense, the implementation of simultaneous saccharification and co-fermentation (SSCF, co-consumption of both glucose and xylose) processes is of upmost importance for this purpose [14]. Nevertheless, the discrepancy in the optimal temperature for *S. cerevisiae* fermentation (30–35 °C) and for saccharolytic enzyme hydrolysis (approximately, 50 °C) poses a major drawback, and in this sense the process would benefit from the use of more thermotolerant strains with xylose consumption abilities. Nonetheless, previous works have shown that yeast strains isolated from different industrial environments respond differently to the same genetic modifications for xylose consumption [14, 26], making the heterogeneity of genetic background an important factor to be taken into account. *S. cerevisiae* PE-2 and CA11 (isolated from a first-generation bioethanol plant and a “chachaça” fermentation process, respectively) were previously engineered for d-xylose consumption with the XR/XDH pathway, and, despite presenting different fermentation profiles, both were capable of ethanol production from different non-detoxified hemicellulosic hydrolysates [14].

Despite the importance of designing robust *S. cerevisiae* strains capable of efficient xylose assimilation, and even though the genetic engineering of *S. cerevisiae* for xylose consumption is extensively studied, few previous works have addressed the comparison of different xylose consumption pathways [27–29]. In a first study, both pathways were compared in a laboratorial strain using a lignocellulosic hydrolysate, with the XR/XDH pathway resulting in higher ethanol productivity while the *Piromyces* XI expression resulted in higher ethanol yield [27]. Li and collaborators [28] obtained similar results on comparing two non-isogenic engineered yeast strains for xylose consumption (one containing the XR/XDH pathway and the other *Piromyces* XI) in synthetic media. More recently, the expression of a XI from *C. phytofermentans* was compared to the expression of the XR/XDH pathway in an industrial strain and synthetic media, and was found to result in higher ethanol yield with similar xylose consumption abilities [29]. Taking these into account, and due to the reported specificities of each of the pathways, we hypothesized that the simultaneous expression of both XR/XDH and XI could be beneficial for xylose consumption in a lignocellulosic fermentation context. Furthermore, to the best of our knowledge, there is only one report of the simultaneous expression of both xylose consumption pathways (using a XI from bovine rumen metagenome) in *S. cerevisiae*: performed in a laboratorial strain and synthetic media, presenting low xylose consumption rates and ethanol yields and lacking a thorough comparison with the sole expression of each of the pathways [30]. Considering these, and the lack of comparison studies performed in more industrial-relevant conditions, in this work the utilization of XR/XDH and/or XI pathways was compared in the industrial (-derived) strains PE-2∆GRE3 and CA11. The effect of the two pathways, either separately or combined, on xylose consumption and ethanol production was evaluated with different fermentation conditions in synthetic xylose-containing medium and also in corn cob lignocellulosic hydrolysate.

## Results

### Effect of different xylose metabolic pathways on the xylose fermentation capacity of *S. cerevisiae* PE-2∆GRE3 and CA11

#### Evaluation of kinetic profiles during xylose fermentation

**Fig. 1:**
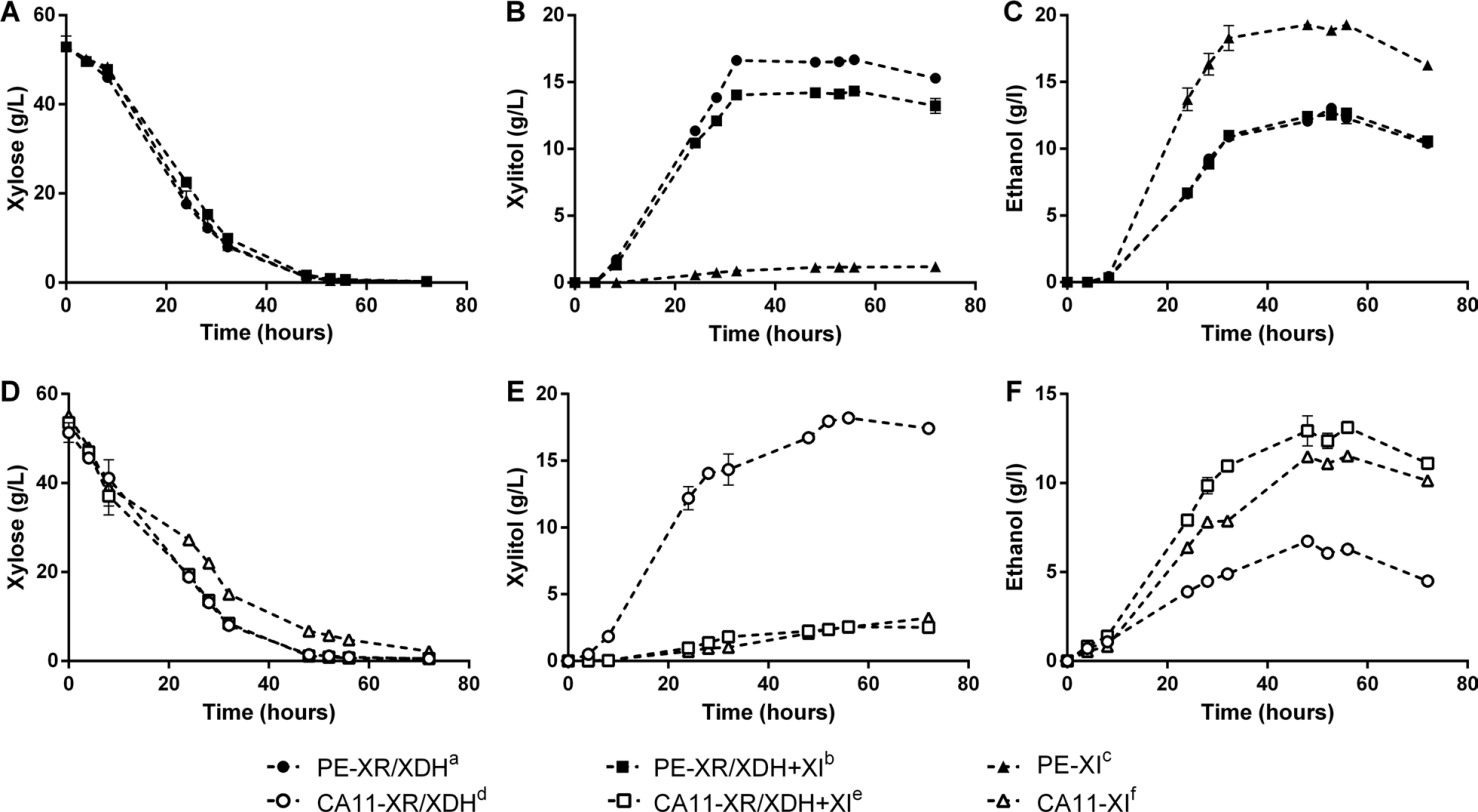
Fermentation profiles in YPX medium at 30 °C. Xylose (**A**, **D**), xylitol (**B**, **E**) and ethanol (**C**, **F**). Xylose: d,f*; e,f*. Xylitol: a,b***; a,c****; b,c****; d,f****; e,f****. Ethanol: a,c***; b,c***; d,e****; d,f***; e,f**

To evaluate the effect of expressing different xylose consumption metabolic pathways, the different constructed strains were characterized for their capacity to metabolize and convert xylose during cotton stopper fermentation in synthetic medium (Fig. 1, Table 1). As seen in Fig. 1A, the strains constructed from PE-2∆GRE3 show similar xylose consumption profiles (at a 95% confidence level); however, the PE-2∆GRE3 containing only the XI pathway was capable of producing higher ethanol concentrations than the strains containing the XR/XDH pathway (Fig. 1C, *P* < 0.001), reaching at 24 h of fermentation an ethanol yield from xylose of 0.401 g/g (corresponding to 79% of the theoretical maximum). On the other hand, the strains expressing the XR/XDH (alone or together with the XI pathway) accumulated higher quantities of xylitol (Fig. 1B, *P* < 0.0001) with yields superior to 0.3 g/g at 24 h of fermentation, while the xylitol yield of the PE-XI strain was lower than 0.02 g/g (Table 1). Regarding the CA11-derived strains, the one containing only the XI pathway presented a slightly lower xylose consumption capacity than the strains with the XR/XDH pathway (Fig. 1D, *P* < 0.05). Nevertheless, the strains expressing the XI pathway, CA11-XR/XDH + XI and CA11-XI, produced higher ethanol titers during the fermentation (Fig. 1F, *P* < 0.0001), both presenting similar ethanol yields (Table 1), while the CA11-XR/XDH strain accumulated higher levels of the by-product xylitol (Fig. 1E, *P* < 0.0001). At the end of fermentation, the glycerol accumulation in the medium was inferior to 1 g/L for all strains. Additionally, the use of cotton stoppers allowed some oxygenation of the medium, and consequently higher amounts of xylose were used for biomass production (in comparison with oxygen-deprived conditions) resulting in lower ethanol yields (with the strain with the highest fermentation performance reaching 79% of the theoretical yield) (Table 1).

**Table 1:** Fermentation parameters in YPX medium at 30 °C

Strain	Xylose_i_ (g/L)	Xylose_f_ (g/L)	Ethanol_max_ (g/L)	Xylitol_max_ (g/L)	*Y*_et/xyl_24 h (g/g)	*Y*_xyol/xyl_24 h (g/g)	DCW (g/L)
*PE-2∆GRE3*							
XR/XDH^a^	52.9 ± 2.5	0.205 ± 0.001	13.0 ± 0.3	16.7 ± 0.1	0.189 ± 0.020	0.324 ± 0.029	11.1 ± 0.8
XR/XDH + XI^b^	52.9 ± 2.5	0.235 ± 0.001	12.7 ± 0.0	14.3 ± 0.2	0.222 ± 0.012	0.346 ± 0.022	13.8 ± 2.3
XI^c^	52.9 ± 2.5	0.215 ± 0.031	19.3 ± 0.4	1.19 ± 0.04	0.401 ± 0.031	0.0167 ± 0.0002	10.8 ± 0.5
*CA11*							
XR/XDH^d^	51.3 ± 2.2	0.543 ± 0.010	6.73 ± 0.19	18.2 ± 0.2	0.120 ± 0.002	0.376 ± 0.014	14.1 ± 0.5
XR/XDH + XI^e^	53.6 ± 1.0	0.556 ± 0.008	13.1 ± 0.3	2.55 ± 0.14	0.232 ± 0.004	0.0287 ± 0.0002	13.1 ± 0.8
XI^f^	55.0 ± 0.4	2.16 ± 0.06	11.5 ± 0.0	3.23 ± 0.05	0.230 ± 0.005	0.0261 ± 0.0008	12.1 ± 0.0

Fermentation time was 72 h. Xylose_*i*_ and Xylose_*f*_ are the xylose concentrations at the initial and final time of fermentation, respectively. Ethanol_max_ is the maximal ethanol concentration reached during the experimental time frame. Xylitol_max_ is the maximal xylitol concentration reached during the experimental time frame. *Y*_et/xyl_24 h is the ethanol yield from xylose calculated at 24 h of fermentation. *Y*_xyol/xyl_24 h is the xylitol yield from xylose calculated at 24 h of fermentation. DCW is the dry cell weight at final time. Xylose_f_: d,f***; e,f***. Ethanol_max_: a,c***; b,c***; d,e***; d,f***; e,f*. Xylitol_max_: a,b**; a,c****; b,c****; d,e****; d,f****. Y_et/xyl_24 h: a,c*; b,c*; d,e***; d,f***. Y_xyol/xyl_24 h: a,c**; b,c**; d,e***; d,f***. DCW: b,c*

#### Evaluation of xylose fermentation capacity in oxygen-deprived conditions at 30 and 40 °C

**Fig. 2:**
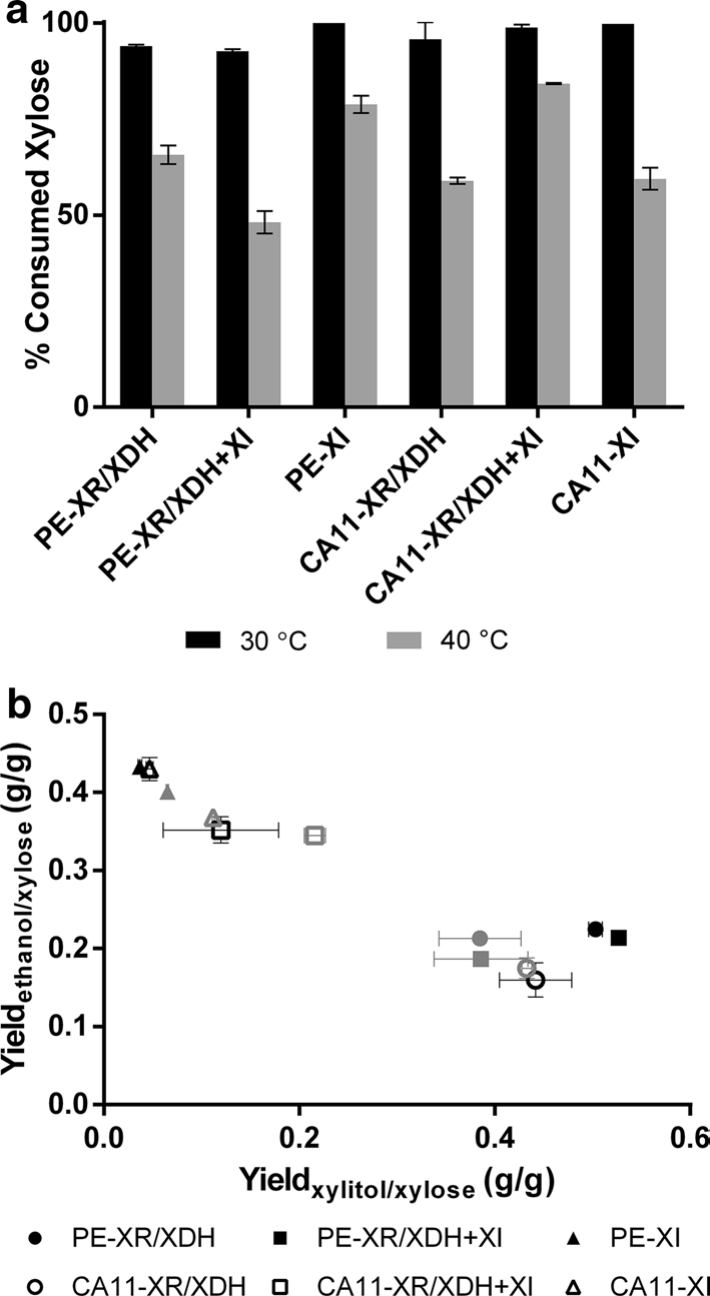
Fermentation parameters in YPX medium at 30 °C and 40 °C in oxygen-deprived conditions. Percentage of xylose consumed at the end of fermentation (120 h) (**a**) and ethanol and xylitol yields from xylose (**b**)

To further evaluate the fermentative capacity of these strains, they were tested in oxygen-deprived conditions to potentiate ethanol production, and also at higher temperature (40 °C) to evaluate their potential in more demanding ethanol fermentation processes, such as simultaneous saccharification and fermentation (SSF) (Fig. 2, Table 2). As previously observed at 30 °C, among the PE-2∆GRE3-derived strains, the strain expressing solely the XI pathway presented a higher ethanol titer and yield from xylose (Fig. 2b, Table 2, *P* < 0.0001), while the strains containing the XR/XDH pathway presented xylitol yields ca. 6 times higher than the PE-XI strain (Fig. 2b, Table 2, *P* < 0.0001). Regarding the strains derived from CA11, the one with sole expression of the XI pathway also reached a higher ethanol concentration (Table 2, *P* < 0.05); as already observed previously (Table 1), CA11-XR/XDH showed higher xylitol accumulation capacity (Table 2, *P* < 0.05). It should be noted that the strains with the highest ethanol production ability were also the ones capable of consuming higher xylose quantities during the time course of fermentation (~ 120 h) at 30 °C (Fig. 2, Table 2). The decrease in oxygen availability increased the amount of ethanol produced by the strains solely containing the XI pathway (yield of ethanol from xylose ~ 0.43 g/g) and by the CA11 strain with both pathways (Fig. 2b, Table 2). However, for the strains that have previously shown a higher potential for xylitol accumulation (PE-XR/XDH, PE-XR/XDH + XI and CA11-XR/XDH, Fig. 1, Table 1), the oxygen-deprived conditions did not improve ethanol production but instead greatly increased the yield of the by-product xylitol (Fig. 2b, Table 2).

**Table 2:** Fermentation parameters in YPX medium at 30 and 40 °C in oxygen-deprived conditions

Strain	Temp (°C)	Xylose_*i*_ (g/L)	Xylose_*f*_ (g/L)	Ethanol_*f*_ (g/L)	Xylitol_*f*_ (g/L)	Glycerol_*f*_ (g/L)	*Y*_et/xyl_ (g/g)	*Y*_xyol/xyl_ (g/g)	DCW (g/L)
*PE-2∆GRE3*	30								
XR/XDH^a^		51.7 ± 0.1	3.11 ± 0.13	10.9 ± 0.0	24.4 ± 0.3	1.44 ± 0.00	0.225 ± 0.002	0.503 ± 0.007	5.78 ± 0.53
XR/XDH + XI^b^		51.7 ± 0.1	3.82 ± 0.20	10.2 ± 0.1	25.3 ± 0.2	0.921 ± 0.007	0.214 ± 0.002	0.527 ± 0.003	5.43 ± 0.34
XI^c^		51.7 ± 0.1	0.00 ± 0.00	22.4 ± 0.1	1.91 ± 0.10	3.24 ± 0.27	0.433 ± 0.004	0.037 ± 0.002	5.27 ± 0.40
*CA11*									
XR/XDH^d^		48.3 ± 0.6	2.06 ± 1.51	7.38 ± 0.87	20.4 ± 1.3	1.11 ± 0.41	0.160 ± 0.022	0.442 ± 0.037	10.35 ± 3.55
XR/XDH + XI^e^		48.3 ± 0.6	0.574 ± 0.249	16.8 ± 0.5	5.67 ± 2.71	0.845 ± 0.265	0.352 ± 0.017	0.120 ± 0.059	10.05 ± 2.45
XI^f^		48.3 ± 0.6	0.135 ± 0.005	20.7 ± 0.46	2.26 ± 0.14	3.50 ± 0.05	0.430 ± 0.015	0.0469 ± 0.0036	6.55 ± 0.15
*PE-2∆GRE3*	40								
XR/XDH^a^		52.0 ± 0.8	17.8 ± 0.6	7.28 ± 0.43	13.1 ± 0.9	1.17 ± 0.01	0.213 ± 0.004	0.385 ± 0.042	1.60 ± 0.10
XR/XDH + XI^b^		52.0 ± 0.8	27.0 ± 1.5	4.67 ± 0.04	9.61 ± 0.94	1.15 ± 0.06	0.187 ± 0.007	0.386 ± 0.048	1.10 ± 0.10
XI^c^		52.0 ± 0.8	11.0 ± 0.7	16.4 ± 0.3	2.67 ± 0.04	3.51 ± 0.01	0.401 ± 0.006	0.0651 ± 0.0012	1.40 ± 0.10
*CA11*									
XR/XDH^d^		48.3 ± 0.6	19.9 ± 0.5	4.98 ± 0.35	12.3 ± 0.2	1.93 ± 0.03	0.175 ± 0.013	0.433 ± 0.005	3.65 ± 0.15
XR/XDH + XI^e^		48.3 ± 0.6	7.63 ± 0.14	14.1 ± 0.1	8.79 ± 0.34	2.07 ± 0.01	0.345 ± 0.006	0.216 ± 0.011	4.00 ± 0.80
XI^f^		48.3 ± 0.6	19.6 ± 0.7	10.6 ± 0.3	3.21 ± 0.17	4.42 ± 0.16	0.368 ± 0.008	0.112 ± 0.001	3.45 ± 0.05

Fermentation time was 120 h. Xylose_*i*_ and Xylose_*f*_ are the xylose concentrations at the initial and final time of fermentation, respectively. Ethanol_*f*_, Xylitol_*f*_ and Glycerol_f_ are the ethanol, xylitol and glycerol concentrations at the final time, respectively. *Y*_et/xyl_ is the ethanol yield from xylose calculated at the final time of fermentation. *Y*_xyol/xyl_ is the xylitol yield from xylose calculated at the final time of fermentation. DCW is the dry cell weight at final time. 30 °C: Xylose_f_: a,c**; b,c***. Ethanol_f_: a,b*; a,c****; b,c****; d,e**; d,f**; e,f*. Xylitol_f_: a,c****; b,c****; d,e*; d,f*. Glycerol_f_: a,c**; b,c**; d,f*; e,f*. Y_et/xyl_24 h: a,c***; b,c***; d,e**; d,f**. Y_et/xyl_: a,c****; b,c****; d,e*; d,f**. Y_xyol/xyl_: a,c****; b,c****; d,e*; d,f*0.40 °C: Xylose_f_: a,b*; a,c*; b,c**; d,e**; e,f**. Ethanol_f_: a,b*; a,c***; b,c***; d,e***; d,f**; e,f**. Xylitol_f_: a,c**; b,c*; d,e**; d,f***; e,f**. Glycerol_f_: a,c****; b,c****; d,f***; e,f***. Y_et/xyl_24 h: a,c**; b,c**; d,e***; d,f**; e,f*. Y_et/xyl_: a,c***; b,c***; d,e**; d,f**. Y_xyol/xyl_: a,c*; b,c*; d,e***; d,f****; e,f**

Considering the fermentations at 40 °C, it is clear that the higher temperature severely affected the performance of all strains, decreasing xylose consumption and ethanol production, as well as biomass growth (Fig. 2a, Table 2). Comparing the PE-2∆GRE3-derived strains in terms of xylose consumption, the PE-XI presented the highest percentage of xylose consumed (79%, Fig. 2a) at the end of fermentation (120 h), while among the strains with CA11 background the one expressing both pathways reached a higher percentage of xylose consumption (84%, Fig. 2a). Nevertheless, the ethanol yields from xylose were not severely affected by the increase in temperature (Fig. 2b, Table 2), with the PE-XI, CA11-XR/XDH + XI and CA11-XI strains presenting the highest values (≥ 0.345 g/g). Curiously, these strains with higher ethanol yields accumulated also higher xylitol quantities at 40 °C than at 30 °C (Table 2). Additionally, in all fermentations, at both temperatures, the strains with sole expression of the XI pathway (PE-XI and CA11-XI) accumulated higher quantities of the by-product glycerol, with the CA11-XI strain accumulating 0.154 g of glycerol per gram of xylose consumed at 40 °C (Table 2, *P* < 0.05).

#### Comparison of enzyme activities in cell extracts of xylose fermentations in oxygen-deprived conditions at 30 and 40 °C

**Fig. 3:**
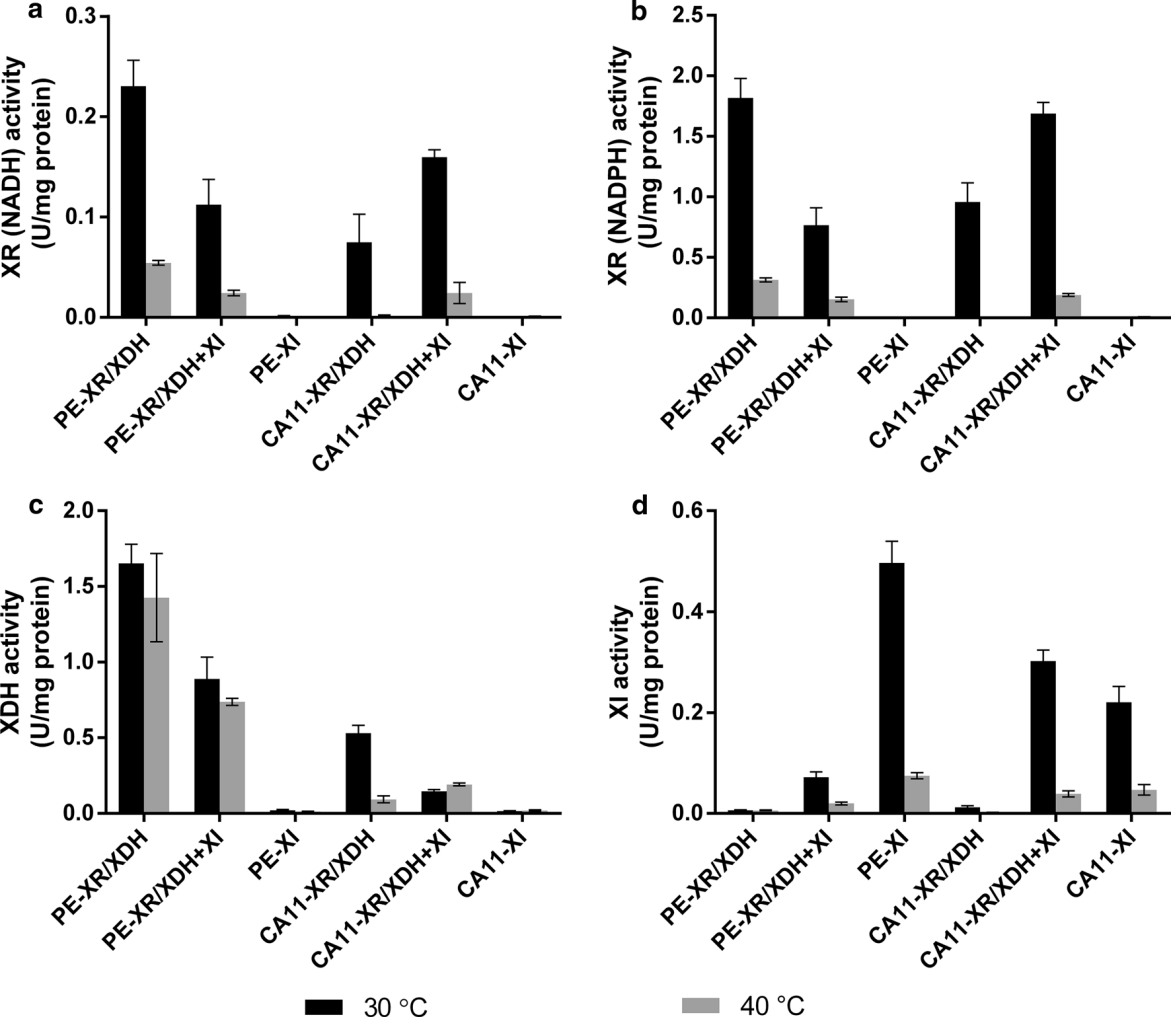
Enzymatic activities of XR with NADH (**a**), XR with NADPH (**b**), XDH (**c**) and XI (**d**). Biomass was collected at 24 h of fermentation in YPX medium at 30 °C and 40 °C in oxygen-deprived conditions

To fully understand the role played by the different pathways in xylose metabolism of each strain, the level of activity of the XR, XDH and XI enzymes was evaluated in yeast cell extracts after 24 h of xylose fermentation at 30 and 40 °C (Fig. 3). At 30 °C, the PE-XR/XDH showed the highest activity among all the strains of the XR (both using NADH and NADPH cofactors) and XDH enzymes (Fig. 3a–c), while the PE-XI strain showed the highest activity of XI enzyme (Fig. 3d). The PE-XR/XDH + XI presented XR and XDH activity levels more than twofold lower than those presented by the PE-XR/XDH strain. Additionally, the XI activity level in this strain was considerably lower (0.07 U/mg of protein) than that from the PE-XI strain (0.50 U/mg of protein) and from the other CA11 strains containing this enzyme (Fig. 3d). Regarding the CA11-derived strains, the XR activity was higher in the strain with both pathways than in the one solely expressing XR/XDH (Fig. 3a, b), while the XDH activity at 30 °C was higher in the CA11-XR/XDH strain (Fig. 3c). The CA11 strain with both pathways also presented a slightly higher XI activity than the CA-XI strain (Fig. 3d) at 30 °C. It should also be noted that while the XR gene introduced in these strains has a mutation for NADH-preference, the activity of the XR enzyme is still much higher (~ 10 times) when using the cofactor NADPH (Fig. 3a, b). Also, and as expected, the strains without heterologous expression of an enzyme presented only residual levels of the corresponding enzymatic activity. With the increase of temperature to 40 °C, the activity levels of the enzymes were severely decreased (Fig. 3), with the exception of the XDH activity in the strains PE-XR/XDH, PE-XR/XDH + XI and CA11-XR/XDH + XI (Fig. 3c).

### Effect of different metabolic pathways on the fermentation capacity of *S. cerevisiae* PE-2∆GRE3 and CA11 in corn cob hydrolysate

#### Evaluation of fermentation capacity in corn cob hydrolysate

**Fig. 4:**
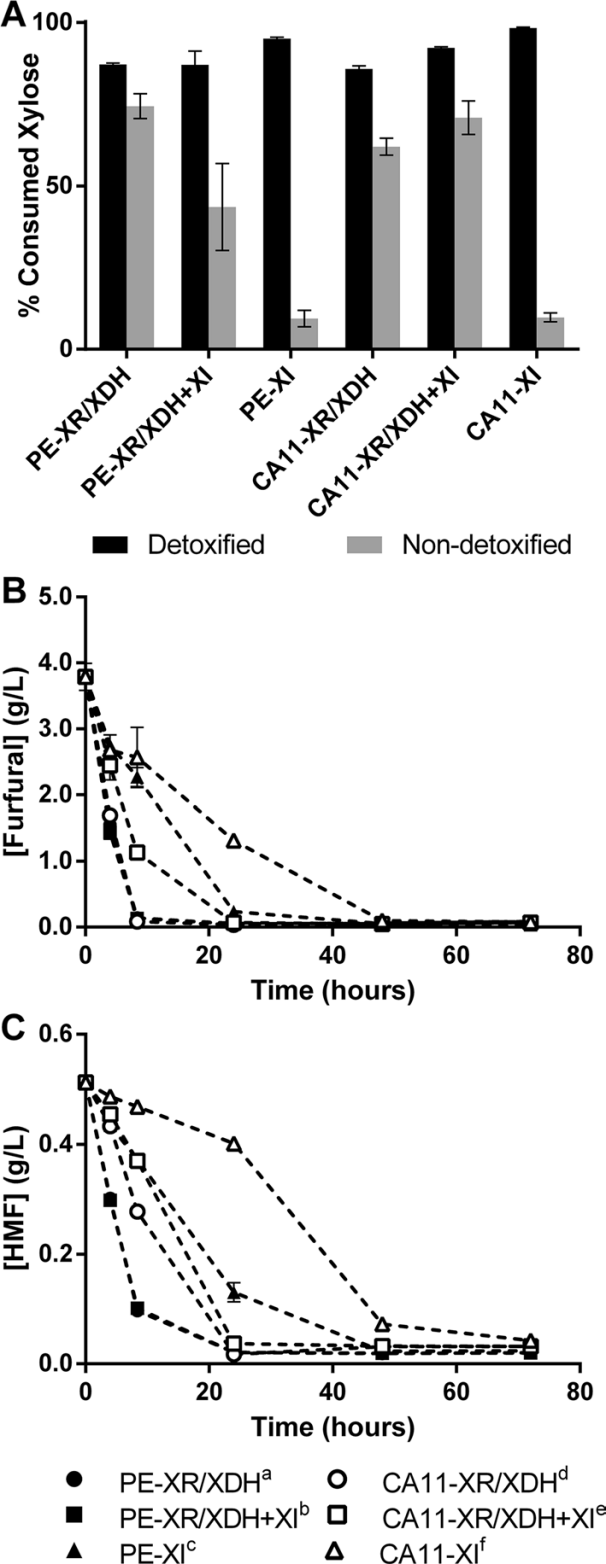
Percentage of consumed xylose in corn cob hydrolysates at 30 °C in oxygen-deprived conditions. Xylose was quantified at the final time of fermentation in detoxified and non-detoxified hydrolysates. Fermentation time course was 73 and 96 h for detoxified and non-detoxified hydrolysate, respectively (**A**). Detoxification profiles of the different strains in YPX medium supplemented with 4 g/L of furfural and 0.5 g/L of HMF (**B**, **C**). Furfural: a,c***; b,c***; d,e*; d,f****; e,f**. HMF: a,c****; b,c****; d,e**; d,f****; e,f****

Corn cob is a lignocellulosic biomass with a high percentage of xylan, which when submitted to appropriate pre-treatment and hydrolysis results in a xylose-enriched liquor. In this sense, this biomass was selected to test the performance of these xylose-consuming strains in lignocellulosic hydrolysates. After treatment, the corn cob hydrolysate comprised per liter ca. 28.3 g of xylose, 1.15 g of glucose, 4.36 g of acetic acid, 0.170 g of HMF and 1.36 g of furfural. Considering the negative effect played by the lignocellulose-derived inhibitors, these strains were tested both with detoxified (Fig. 4A) and non-detoxified (Fig. 4A) corn cob hydrolysate. Detoxified hydrolysate contained per liter ca. 26.74 g of xylose, 2.45 g of glucose and 0.43 g of acetic acid.

The fermentative performance of the different strains with the detoxified corn cob hydrolysate (Fig. 4A, Table 3) were similar to that observed with synthetic medium at 30 °C (Fig. 2a, Table 2), for the strains solely expressing the XI pathway (PE-XI and CA11-XI) and consuming higher amounts of xylose. Similarly, the PE-XI and CA11-XI strains were the ones presenting higher ethanol titers, reaching yields of 0.44 g of ethanol per gram of consumed sugars (Table 3, *P* < 0.01), while the PE-XR/XDH, PE-XR/XDH + XI and CA11-XR/XDH accumulated higher amounts of xylitol, with yields higher than 0.427 g of xylitol per gram of xylose (Table 3, *P* < 0.01). When comparing the strains with both pathways, it should be noted that the CA11-derived strain presented higher ethanol yield and lower xylitol accumulation levels than the PE-derived strain (Table 3), as already observed in synthetic medium (Table 2, Fig. 2b).

**Table 3:** Fermentation parameters in corn cob hydrolysate (detoxified and non-detoxified) at 30 °C in oxygen-deprived conditions

Strain	Hydrolysate	Xylose_*i*_ (g/L)	Xylose_f_ (g/L)	Ethanol_*f*_ (g/L)	Xylitol_f_ (g/L)	Glycerol_*f*_ (g/L)	*Y*_et/sug_ (g/g)	*Y*_xyol/xyl_ (g/g)	DCW (g/L)
*PE-2∆GRE3*	Detoxified corn cob								
XR/XDH^a^		26.7	3.43 ± 0.07	6.09 ± 0.05	10.3 ± 0.1	0.821 ± 0.013	0.261 ± 0.001	0.442 ± 0.005	2.50 ± 0.10
XR/XDH + XI^b^		26.7	3.46 ± 0.79	6.06 ± 0.28	9.91 ± 0.46	2.57 ± 1.82	0.260 ± 0.003	0.427 ± 0.034	2.80 ± 0.20
XI^c^		26.7	1.31 ± 0.07	11.2 ± 0.3	0.714 ± 0.029	0.880 ± 0.384	0.439 ± 0.012	0.028 ± 0.001	3.80 ± 0.50
*CA11*									
XR/XDH^d^		26.7	3.79 ± 0.17	4.93 ± 0.05	10.6 ± 0.1	1.47 ± 0.13	0.215 ± 0.004	0.462 ± 0.007	2.50 ± 0.10
XR/XDH + XI^e^		26.7	2.07 ± 0.05	9.23 ± 0.07	4.94 ± 0.05	0.571 ± 0.004	0.374 ± 0.004	0.200 ± 0.002	3.25 ± 0.15
XI^f^		26.7	0.438 ± 0.035	11.6 ± 0.2	0.958 ± 0.053	1.47 ± 0.03	0.440 ± 0.008	0.036 ± 0.002	4.60 ± 0.30
*PE-2∆GRE3*	Non-detoxified corn cob								
XR/XDH^a^		28.0 ± 0.1	7.16 ± 0.74	6.29 ± 0.80	5.46 ± 0.54	1.55 ± 0.05	0.272 ± 0.025	0.264 ± 0.036	1.77 ± 0.03
XR/XDH + XI^b^		28.0 ± 0.1	15.8 ± 2.6	4.04 ± 0.80	3.06 ± 0.09	0.956 ± 0.069	0.279 ± 0.004	0.262 ± 0.050	1.43 ± 0.17
XI^c^		28.0 ± 0.1	25.3 ± 0.5	1.16 ± 0.15	0.275 ± 0.026	0.393 ± 0.076	0.243 ± 0.057	0.107 ± 0.011	1.07 ± 0.07
*CA11*									
XR/XDH^d^		28.6 ± 0.0	10.9 ± 0.5	4.83 ± 0.35	5.15 ± 0.04	2.03 ± 0.13	0.244 ± 0.008	0.291 ± 0.006	2.23 ± 0.03
XR/XDH + XI^e^		28.6 ± 0.0	8.33 ± 1.05	8.55 ± 0.22	1.44 ± 0.08	1.20 ± 0.22	0.384 ± 0.013	0.0716 ± 0.0074	2.40 ± 0.00
XI^f^		28.6 ± 0.0	25.8 ± 0.3	1.34 ± 0.10	0.275 ± 0.047	0.608 ± 0.210	0.279 ± 0.013	0.0977 ± 0.0073	1.60 ± 0.27

Fermentation time course was 73 and 96 h for detoxified and non-detoxified hydrolysate, respectively. Xylose_*i*_ and Xylose_f_ are the xylose concentrations at the initial and final time of fermentation, respectively. Ethanol_*f*_, Xylitol_f_ and Glycerol_f_ are the ethanol, xylitol and glycerol concentrations at the final time, respectively. *Y*_et/sug_ is the ethanol yield from initial sugar calculated at final time of fermentation. Y_xyol/xyl_ is the xylitol yield from xylose calculated at the final time of fermentation. DCW is the dry cell weight at final time. Detoxified Corn Cob—Xylose_f_: d,e**; d,f***; e,f**. Ethanol_f_: a,c**; b,c**; d,e***; d,f****; e,f**. Xylitol_f_: a,c***; b,c***; d,e****; d,f****; e,f****. Glyferol_*f*_: d,e**; e,f**. Y_et/xyl_: a,c***; b,c***; d,e***; d,f***; e,f**. Y_xyol/xyl_: a,c**; b,c**; d,e****; d,f****; e,f***. DFW: d,f*; e,f*. Non-detoxified Corn Cob- Xylose_f_: a,c**; b,c*; d,f**; e,f***. Ethanol_f_: a,c*; d,e**; d,f**; e,f***. Xylitol_f_: a,b*; a,c**; b,c*; d,e****; d,f****; e,f**. Glycerol_f_: a,b*; a,c**; b,c*; d,f*. Y_et/xyl_: d,e**; e,f*. Y_xyol/xyl_: d,e***; d,f***. DCW: a,c*

On the other hand, the presence of inhibitory compounds such as acetic acid, furfural and HMF in the non-detoxified corn cob hydrolysate severely affected the fermentative capacity of all the strains, reducing xylose consumption and ethanol production (Fig. 4A, Table 3). The strains only containing the XI pathway, PE-XI and CA11-XI, were essentially incapable of xylose consumption (less than 10% of initial xylose in 96 h of fermentation) (Fig. 4A), producing only residual amounts of ethanol (Table 3). From the PE-2∆GRE3-derived strains, the one with only the XR/XDH pathway consumed higher amounts of xylose during the time course of fermentation (Fig. 4A, *P* < 0.01), but produced low amounts of ethanol while accumulating xylitol (yields of 0.272 and 0.264 g/g, respectively) (Table 3). On the other hand, the CA11-XR/XDH + XI strain, which consumed approximately the same amount of xylose as PE-XR/XDH, presented the highest ethanol yield (0.384 g/g) of all the strains tested in non-detoxified corn cob hydrolysate (an improvement of more than 38% in comparison with the other strains) (Table 3, *P* < 0.05) with low xylitol production (corresponding to a xylitol yield of 0.0716 g/g) (Table 3).

#### Evaluation of furfural and HMF detoxification capacity

To further elucidate the association between the xylose-consuming pathway and the yeast capacity for detoxification of furan compounds, the different strains were used for fermentation of xylose-containing synthetic media supplemented with 4 g/L of furfural and 0.5 g/L of HMF (Fig. 4B, C). For both furfural and HMF detoxification, the strains expressing the XR/XDH pathway showed improved capacity when compared to the ones with high activity of the XI enzyme (Fig. 4B, C, *P* < 0.05). However, while the strains without or with low XI activity were capable of furfural depletion in approximately 8 h, the CA11-XR/XDH + XI strain only completely detoxified furfural after 24 h (Fig. 4B).

## Discussion

The economically viable production of second-generation bioethanol must comprise an effective conversion of the xylose present in the lignocellulosic biomass. Normally, xylose consumption in *S. cerevisiae* is obtained by the heterologous expression of the XR/XDH or the XI pathways. While several xylose-consuming *S. cerevisiae* strains have been constructed using either strategy, the reported ethanol yields from xylose are still far below the theoretical yield [31–34]. On the other hand, there is a lack of consensus in the few studies comparing the use of the different xylose-consuming pathways: while all describe the strains containing the XI pathway as capable of reaching higher ethanol yields than the ones containing the XR/XDH pathway, the XI role in xylose consumption rates and ethanol productivity is not clear [27–29]. Considering the role that different yeast backgrounds may play in the final xylose consumption capacity, in this work two different industrial *S. cerevisiae* strains were used to systematically compare the efficiency of the two pathways, and also of the combination of both pathways, for xylose consumption in the absence or presence of lignocellulosic-derived inhibitors.

The first evaluation of the performance of the different strains, in synthetic media and in the presence of oxygen, revealed a clear advantage of the strains expressing XI for ethanol production, while the strains containing the XR/XDH pathway accumulated higher xylitol levels. Nevertheless, these conditions favored biomass production, resulting in lower ethanol yields. As expected, when tested in oxygen-deprived conditions, the strains containing solely the XI pathways achieved higher ethanol yields, of ca. 0.43 g/g of xylose, which are in accordance with the values previously obtained in synthetic xylose medium using an industrial *S. cerevisiae* strain expressing the same XI [20]. Interestingly at 30 °C, the strains with higher XI activity (PE-XI, CA11-XR/XDH + XI and CA11-XI) consumed more xylose during the time course of fermentation. The source of the XI enzyme may explain this difference in performance, as in previous comparative investigations, using XI from the fungus *Piromyces* sp. in *S. cerevisiae* strains, the XI-containing strains showed lower xylose consumption rates than the ones containing XR/XDH [27, 28], while a more recent comparative study using the XI from the anaerobic bacterium *C. phytofermentans* (also used in this work) showed similar xylose consumption profiles [29]. As expected, the strains with low XI activity levels (PE-XR/XDH, PE-XR/XDH + XI and CA-XR/XDH), which metabolize xylose mainly through the XR/XDH pathway, presented higher xylitol yields. In fact, fermentation in oxygen-deprived conditions also resulted in higher accumulation of xylitol in the XR/XDH-utilizing strains, as the decrease in aerobic respiration reduces the oxidation of NADH, and the consequent low NAD^+^/NADH ratio favors xylitol production [35]. It should be noted that even with the deletion of the *GRE3* gene, the PE-2 strain containing the XR/XDH pathway produced similar, and sometimes higher, xylitol levels than the CA11-derived strain, as a result of the already described natural predisposition of PE-2 for accumulation of this compound [13, 14, 36]. Moreover, the differences between the strains PE-XR/XDH + XI and CA11-XR/XDH + XI clearly corroborate the significant role played by the XR/XDH pathway in xylitol accumulation and the association between the XI pathway and higher ethanol production: in the CA11-derived strain, there is an increase in the XI activity when both pathways are used, achieving higher ethanol yields, while in the PE-XR/XDH + XI strain only residual levels of XI activity could be observed, resulting in xylitol yields similar to those in the PE-2∆GRE3 strain containing only the XR/XDH pathway.

The performance of the xylose-consuming strains was also evaluated in fermentations at higher temperature, which, as expected, resulted in an accentuated reduction in their xylose consumption and ethanol and xylitol production capacity, which is in accordance with the significant decrease in the activity level of almost all xylose consumption enzymes at 40 °C. Nevertheless, and as observed at 30 °C, the PE-XI and CA11-XI strains presented higher ethanol yields than the XR/XDH containing strains. Interestingly, the PE-2∆GRE3-derived strains containing the XR/XDH pathway were able to maintain a high activity level of the XDH enzyme at 40 °C, probably explaining their reduced xylitol accumulation at this temperature. The *S. cerevisiae* CA11 strain has previously been reported to have thermotolerant fermentation capacity [14, 37], and despite the fact that the CA11-derived strains generally did not show a better performance when compared with the PE-2∆GRE3-derived strains, the CA11-XR/XDH + XI strain showed the highest xylose consumption capacity among all the strains at 40 °C.

**Fig. 5:**
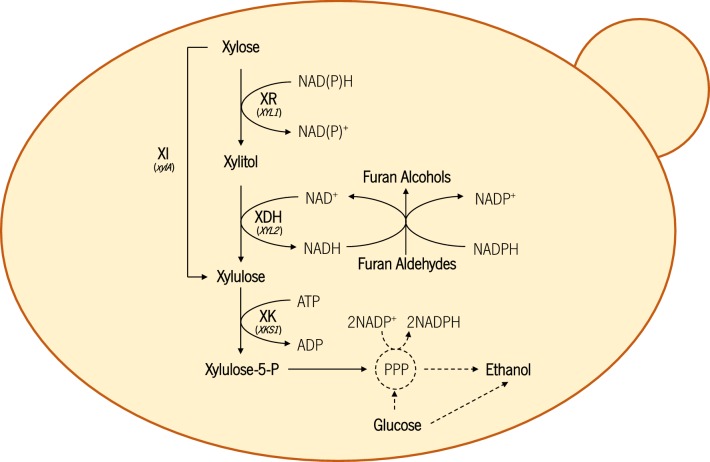
Schematic representation of different xylose-consuming pathways, glucose metabolic pathway and furan detoxification in *S. cerevisiae*

To test the applicability of these strains in conditions more close to real industrial conditions, they were tested in hemicellulosic corn cob hydrolysate. When firstly submitted to fermentation with detoxified hydrolysate, performance of the strains was similar to that obtained in synthetic media: higher ethanol yields obtained from the strains with high XI activity, and more xylitol accumulation from the strains mainly using the XR/XDH pathway. In non-detoxified hydrolysate, and despite the robust characteristics of the industrial *S. cerevisiae* chassis used, the presence of inhibitors such as acetic acid, furfural and HMF greatly hampered the fermentative capacity of all strains, but mainly of the strains containing only the XI pathway. The lack of the XR/XDH pathway may explain the low xylose consumption and lower level of ethanol production presented by the PE-XI and CA11-XI strains. In fact, the XR enzyme from *P. stipitis* has been described as having the capacity to convert HMF into less toxic compounds [38], and in this work, the presence of the XR/XDH pathway was found to greatly increase the detoxification rate, not only of HMF, but also of furfural. Additionally, the presence of furfural has been described previously as being advantageous for ethanol production from xylose through the XR/XDH pathway, as its NADH-dependent detoxification regenerates NAD^+^, relieving the redox imbalance and reducing xylitol accumulation [39, 40]. In turn, the conversion of xylitol into xylulose regenerates NADH that can be used for further detoxification. In this sense, as a result of the redox equilibrium between furan bioconversion and the XR/XDH pathway (Fig. 5), the strains containing this pathway were capable of xylose fermentation in the non-detoxified hydrolysate. This effect can be observed clearly in the furfural detoxification pattern of the CA11-XR/XDH + XI strain (which has high enzymatic activity of both the XR/XDH and XI pathways): as xylose conversion is divided between the two pathways, less NADH is regenerated by the XR/XDH pathway, resulting in slower furfural detoxification than in the strain mainly using the XR/XDH pathway, but still faster than in the one containing only the XI pathway. In fact, the CA11-XR/XDH + XI strain achieved the highest ethanol production and yield, indicating a clear advantage of expressing both pathways for fermentation of non-detoxified hydrolysates: XR/XDH which facilitates inhibitor detoxification with concomitant decreased xylitol accumulation; and XI, which also reduces xylitol accumulation and allows high conversion yields of xylose into ethanol. Accordingly, the strain PE-XR/XDH + XI, despite presenting high detoxification capacity (derived from the presence of the XR/XDH pathway), presents a low enzymatic activity of XI, which results in higher xylitol accumulation, lower xylose consumption and higher ethanol titers in non-detoxified hydrolysate (in comparison with CA11-XR/XDH + XI). However, the accumulation of xylitol by this yeast, despite being considerably smaller than in the absence of inhibitors, may still prevent the attainment of higher ethanol yields. Regarding xylitol accumulation, the use of XI from *C. phytofermentans* clearly shows an advantage, as this enzyme was found to be far less inhibited by xylitol than other XIs already expressed in *S. cerevisiae* [20]. Also worth mentioning is the fact that, despite the reported role of *GRE3* in detoxification of lignocellulosic-derived aldehydes, such as furfural, HMF and methylglyoxal [41, 42], the deletion of this gene in the PE-2 strain did not seem to influence its detoxification capacity negatively, as it remained similar, or even superior, to that of the CA11-derived strains.

## Conclusions

In this work, we have compared the performance of two industrial *S. cerevisiae* strains containing XI and/or XR/XDH for xylose consumption and ethanol production in synthetic medium and corn cob hydrolysate. It is, to the best of our knowledge, the first study involving the simultaneous expression of both xylose consumption pathways in comparison with the single expression of one of the pathways. In synthetic medium, the XI-carrying strains showed the highest ethanol titer and yield, with low xylitol production, and a similar pattern was obtained when the strains were tested in detoxified corn cob hydrolysate. In non-detoxified corn cob hydrolysate, the CA11-XR/XDH + XI strain presented the highest ethanol yield, which can be explained by the high enzymatic activity of the XR/XDH and XI pathways, which allows furfural and HMF detoxification and high ethanol production with low xylitol formation. In this sense, a fine-tuning of the expression of the XR/XDH and XI pathways in the same yeast strain, possibly coupled with additional strategies to decrease xylitol accumulation, would be beneficial to increase the efficiency of ethanol production with undetoxified lignocellulosic hydrolysates.

## Methods

### Yeast strains and plasmid constructions

The yeast strains, plasmids and primers used in this study are listed in Table 4. The industrially derived *S. cerevisiae* PE-2∆GRE3 strain (with the *GRE3* deleted to avoid the natural ability of PE-2 for xylitol accumulation) [13] and the industrial isolate CA11, a flocculent strain [43, 44], were used as chassis strains for comparison of the two xylose consumption pathways. The plasmid for simultaneous expression of the XR/XDH and XI pathways was constructed by insertion of the xylA gene from *C. phytofermentans* (with HXT7 promoter and CYC terminator, amplified from pBED-CpXI-NATMX plasmid with the primer pair XI_FW/XI_RV) in the PvuII restriction site of pMEC1049 by in vivo homologous recombination (pMEC1049 + XI). For the sole expression of the XI pathway, the XYL1/XYL2 transcriptional units were removed by in vivo homologous recombination of the pMEC1049 + XI plasmid (digested with EcoNI) with the PGItp-containing fragment (amplified from pYPKa_Z_PGI1tp with the primer pair 577/567), resulting in the plasmid pMEC_XI. The resulting plasmids were extracted and transformed to *Escherichia coli* NZY5α (NZYtech) for propagation and confirmation by restriction analysis, followed by insertion into the PE-2∆GRE3 and CA11 strains using the LiAC/SS carrier DNA/PEG method [45]. The transformants were selected on YPD plates (10 g/L yeast extract, 20 g/L peptone, 20 g/L glucose and 20 g/L agar) containing 300 μg/mL of hygromycin and were further cultured in YPX medium (10 g/L yeast extract, 20 g/L peptone, 20 g/L d-xylose) until capable of xylose consumption. PE-2∆GRE3 and CA11 strains carrying the pMEC1049 plasmid for expression of the XR/XDH pathway have been described previously [13, 14]. The recombinant strains were preserved at 4 °C on YPX plates containing 300 μg/mL of hygromycin to maintain selective pressure for the plasmids.

**Table 4:** Strains, plasmids and primers used in this study

	Relevant features	Source
*S. cerevisiae* strains		
PE-2∆GRE3	*S. cerevisiae* PE-2 (isolated from Brazilian first generation bioethanol plants [44, 46], gre3::natMX4/gre3::kanMX4	Romaní et al. [13]
PE-XR/XDH	PE-2∆GRE3, pMEC1049	Romaní et al. [13]
PE-XR/XDH + XI	PE-2∆GRE3, pMEC1049 + XI	This work
PE-XI	PE-2∆GRE3, pMEC_XI	This work
CA11	*S. cerevisiae* strain isolated from Brazilian “cachaça” fermentation processes	Pereira et al. and Schwan et al. [43, 44]
CA11-XR/XDH	CA11, pMEC1049	Costa et al. [14]
CA11-XR/XDH + XI	CA11, pMEC1049 + XI	This work
CA11-XI	CA11, pMEC_XI	This work
Plasmids		
pMEC1049	pYPK4-TEF1tp-XR(N272D)-TDH3tp-XDH-PGI1tp-XKS1-FBA1tp-TAL1- PDC1tp, HphMX4	Romaní et al. [13]
pBED-CpXI-NATMX	Plasmid containing the *xylA* gene from *Clostridium phytofermentans* with HXT7 promoter and CYC terminator	Brat et al. [20]
pMEC1049 + XI	pMEC1049 containing the *xylA* gene from *C. phytofermentans* with HXT7 promoter and CYC terminator	This work
pYPKa_Z_PGI1tp		Pereira et al. [47]
pMEC_XI	pMEC1049 + XI after removal of XR and XDH coding sequences	This work

Primers	Sequence (5′– > 3′)	
XI_FW	ACGATTTAGGTGACACTATAGAACGCGGCCGCCAGGAGCTCGTAGGAACAA	
XI_RV	GGGGATCCGTCGACCTGCAGCGTACGAAGCTTCAGCCGATCTCCAGCCGAC	
577	GTTCTGATCCTCGAGCATCTTAAGAATTC	
567	GTCGGCTGCAGGTCACTAGTGAG	

### Non-detoxified corn cob hydrolysate

Corn cob was collected, milled and submitted to hydrothermal treatment (autohydrolysis) under non-isothermal conditions (*T*_max_ of 205 °C, corresponding to a severity of 3.85) based on previous works [48, 49]. Autohydrolysis treatment was carried out in a 2 L stainless steel reactor (Parr Instruments Company) equipped with Parr PDI temperature controller (model 4848) at liquid to solid ratio of 8 g distilled water/g of dry corn cob. After autohydrolysis, the resulting solid and liquid phases were separated by filtration. Liquid phase or autohydrolysis liquor was submitted to acid posthydrolysis (1.5% H_2_SO_4_, 165 min at 121 °C) to hydrolyze the oligomers into monomer sugars, based on previously optimized conditions [49]. The composition of non-detoxified hydrolysate (sugars, acetic acid and furan compounds) was determined by HPLC analysis. The hydrolysate was neutralized using CaCO_3_ until pH 5 and sterilized by filtration (0.2 µm).

### Detoxified corn cob hydrolysate

To remove inhibitors (phenolic compounds, weak acids and furans), corn cob hydrolysate was detoxified using activated charcoal and ionic exchange resins. Corn cob hydrolysate was mixed with activated charcoal at liquor to solid ratio of 10 g of hydrolysate per gram of activated charcoal with agitation for 1 h [50] to remove phenolic compounds. After that, the hydrolysate was treated with ion exchange resins as described by Rodríguez-López et al. [50]. The hydrolysate was mixed with Amberlite IR-120 resin in H^+^ form at a ratio 10 g of cationic resin per gram of hydrolysate for 1 h with agitation. Cationic resin was recovered by filtration and the hydrolysate was treated for 2 h under agitation with Mto-Dowex M43 (anionic) resin in OH^−^ form at a ratio 20 g of anionic resin per gram of acetic acid present in the hydrolysate. The detoxified hydrolysate was sterilized by filtration and analyzed by HPLC.

### Inoculum preparation

Yeast cells for inoculation were grown in YPX medium (10 g/L yeast extract, 20 g/L peptone, 20 g/L d-xylose) at 30 °C for 24 h, with orbital shaking (200 rpm). The cell suspension was collected by centrifugation for 5 min (at 4000*g* and 4 °C) and suspended in 0.9% (w/v) sodium chloride solution to obtain a suspension with 200 mg of fresh yeast/mL. The fermentation assays were inoculated with 5 mg of fresh yeast/mL (approximately, 1 mg of dry cell weight (DCW)/mL).

### Fermentation assays

Fermentations were performed in 100 mL Erlenmeyer flasks, with cotton stopper or glycerol lock to prevent the entrance of oxygen (creating oxygen-deprived conditions) [25], and with a working volume of 30 mL and carried out at 30 or 40 °C in an orbital shaker at 150 rpm. Fermentations with cotton stopper were monitored by sample collection for HPLC analysis; oxygen-deprived fermentations were monitored by measurement of mass loss resulting from CO_2_ production (data not show) and samples for HPLC were collected at the end of fermentation. Fermentation media consisted of YPX medium (10 g/L yeast extract, 20 g/L peptone, 50 g/L d-xylose) and corn cob hydrolysate (detoxified and non-detoxified) supplemented with YP (10 g/L yeast extract, 20 g/L peptone) or YPX medium supplemented with 4 g/L of furfural and 0.5 g/L of HMF.

### Enzymatic activities

Cells for enzyme assays were collected at 24 h of fermentation in YPX medium at 30 and 40 °C. Crude cell extracts were prepared with Y-PER reagent (Thermo Fisher Scientific) and the protein content was determined by Bradford assay (Bio-Rad) [51]. XR, XDH and XI enzymatic activities in each of the cell extracts were assayed (in triplicate) by measuring the decrease/increase of NAD(P)H at 30 °C in a reaction mixture using a microplate reader spectrophotometer (at 340 nm). The reaction mixtures (containing appropriate dilutions of cell crude extract) were adapted from previously reported assays and their compositions were as follows: triethanolamine (100 mM, pH 7.0), NADH or NADPH (0.2 mM) and xylose (350 mM) for XR [52]; glycine (100 mM, pH 9.0), MgCl_2_ (50 mM), NAD + (3.0 mM) and xylitol (300 mM) for XDH [52]; Tris–HCl (100 mM, pH 7.5), MgCl_2_ (10 mM), NADH (0.15 mM), sorbitol dehydrogenase 2 U/mL and xylose (500 mM) for XI [53]. Specific activity is expressed as units per milligram of protein (U/mg protein), in which units (U) is defined as micromol NAD(P)H reduced or oxidized per min.

### Determination of fermentation parameters

Ethanol yield from xylose (*Y*_et/*x*_) was calculated as the ratio between the ethanol concentration at a defined time of fermentation and the xylose consumed in that period of time. Ethanol yield from sugars (*Y*_et/sug_) was calculated as the ratio between the ethanol concentration at a defined time of fermentation and the sugar (xylose and glucose) consumed in that period of time. Xylitol yield from xylose (*Y*_xyol/xyl_) was calculated as the ratio between the xylitol concentration at a defined time of fermentation and the xylose consumed in that period of time.

### Analytical methods

Samples from corn cob treatment (non-detoxified and detoxified hydrolysates) and from fermentation assays were analyzed for quantification of glucose, xylose, acetic acid and ethanol by HPLC using a Bio-Rad Aminex HPX-87H column, operating at 60 °C, with 0.005 M H_2_SO_4_ and at a flow rate of 0.6 mL/min. The compounds were detected using a Knauer-IR refractive index detector. Furfural and HMF were quantified by ultra-high-performance liquid chromatography (UHPLC) using a Shimadzu Nexera X2 UHPLC chromatograph equipped with diode array detector (Shimadzu, SPD-M20A). Separation was performed on a reversed-phase Acquity UHPLC BEH C18 column (2.1 mm × 100 mm, 1.7 μm particle size; from Waters) at 40 °C. The flow rate was 0.4 mL/min. The HPLC-grade solvents used were water/formic acid (0.1%) as solvent A and acetonitrile as solvent B. The elution gradient for solvent B was as follows: from 0.0 to 5.5 min at 5%, from 5.5 to 17 min a linear increase to 60%, from 17.0 to 18.5 min a linear increase to 100%, then column equilibration from 18.5 to 30.0 min at 5%.

### Statistical analysis

Statistical analyses were carried out in GraphPad Prism for Windows version 6.01. Differences between strain performance in terms of ethanol and xylitol production, sugar consumption and furan detoxification profiles were tested by repeated measures two-way ANOVA, followed by Tukey post hoc test. Differences in kinetic parameters were determined using repeated measures one-way ANOVA, followed by Tukey post hoc test. Statistical significance was established at *P* < 0.05 for the comparisons and marked by *P <  0.05; **P <  0.01; ***P < 0.001; ****P < 0.0001.

## Abbreviations

XI: xylose isomerase
XR: xylose reductase
XDH: xylose dehydrogenase
HMF: 5-hydroxymethylfurfural
DCW: dry cell weight
SSF: simultaneous saccharification and fermentation
SSCF: simultaneous saccharification and co-fermentation
HPLC: high-performance liquid chromatography
UHPLC: ultra-high-performance liquid chromatography
ANOVA: analysis of variance
